# Management of metastatic renal cell carcinoma – mini review

**DOI:** 10.15586/jkcvhl.2015.28

**Published:** 2015-05-05

**Authors:** Anubha Bharthuar, Himanshu Pandey, Swapan Sood

**Affiliations:** 1 Department of Medical Oncology and Hematology; 2 Department of Urology, Patel Cancer and Superspeciality Hospital, Jalandhar City, Punjab, INDIA.

## Abstract

The management of metastatic renal cell carcinoma (mRCC) has evolved considerably in the last decade. A number of different systemic molecular targeted agents that have been recently approved have improved the survival of patients with mRCC. This mini-review focuses on the implementation of multi-modality therapy in the management of mRCC and the approved indications of the various available novel agents. These novel agents have expanded our armamentarium and improved clinical outcomes of this challenging disease that has considerable biological heterogeneity and clinical variability.

## Introduction

Kidney cancer is the twelfth most common cancer worldwide - 338,000 new cases were diagnosed in 2012 (1). The highest incidence of kidney cancer is seen in Northern America and Europe while the lowest incidence is seen in Africa and Asia. Of these, renal cell carcinoma (RCC) accounts for more than 90% of cases and represents about 2–3% of adult malignancies. Despite recent scientific advances in diagnosis and management almost 25–30% RCC patients are metastatic at diagnosis (2), and another 20–30% of patients with localized disease who undergo nephrectomy develop metastasis, with a median time to relapse of 15–18 months (3).

## Prognostic models

There is considerable variability in the natural history of this disease and several prognostic models have been developed for metastatic RCC (mRCC). These models help clinicians while counselling patients regarding the expected clinical course and facilitate treatment planning.

The most well-known of these is the MSKCC (Memorial Sloan-Kettering Cancer Center) model which has undergone several revisions across the years. The original publication established five prognostic factors to predict survival in patients with mRCC (4). These included poor performance status (Karnofsky score <80), elevated serum lactate dehydrogenase (LDH) level (>1.5 times upper limit of normal), low hemoglobin (less than the lower limit of normal), elevated corrected calcium concentration (>10 g/dL), and lack of prior nephrectomy. The overall survival (OS) in patients with no adverse factors (favorable-risk group), one to two risk factors (intermediate-risk group), and more than three risk factors (poor-risk group) were 20 months, 10 months, and 4 months, respectively. Subsequent review in the interferon (IFN) alpha treated patients identified time from initial RCC diagnosis to start of IFN therapy of less than one year as another indicator of poor prognosis (5).

The MSKCC model was externally validated by the Cleveland Clinic, who additionally identified prior radiotherapy and presence of hepatic, lung, and retroperitoneal nodal metastases to be predictors of poor prognosis (6). In the era of vascular endothelial growth factor (VEGF) targeted therapy, another prognostic model was derived from patients treated with the tyrosine kinase inhibitors (TKIs) sorafenib, Sunitinib, and bevacizumab with IFN (7). This model is known as the Heng’s model or the International Metastatic RCC Database Consortium model. It includes six prognostic factors - hemoglobin less than the lower limit of normal, corrected calcium greater than the upper limit of normal (ULN), Karnofsky performance status less than 80%, time from diagnosis to treatment of less than one year, neutrophils greater than the ULN and platelets greater than the ULN. This model too has been externally validated in another dataset and has significant current utility in stratifying patients of ongoing clinical trials and clinical relevance while prognosticating patients (8).

## Role of surgery

Surgery as an independent therapeutic modality has limited utility in the mRCC setting. However, cytoreductive nephrectomy followed by systemic therapy is still a recommended strategy for patients with resectable disease. The evidence for this strategy stems from two large randomized phase III trials where patients who underwent cytoreductive nephrectomy followed by IFN showed a survival benefit as compared to those who received IFN treatment alone (13.6 months versus 7.8 months; p=0.002) (9). The role of cytoreductive nephrectomy in the molecular therapy era is being studied in a number of prospective clinical trials, both as an upfront treatment strategy (10) or after neoadjuvant molecular targeted therapy (11). A retrospective analysis showed that patients who underwent cytoreductive nephrectomy followed by targeted therapy had prolonged survival as compared to those who received targeted therapy alone (20.6 months versus 9.5 months; p<0.0001). The benefit was incremental as survival time lengthened, but for those with a survival of less than one year the benefit was marginal (12). Appropriate patient selection is therefore crucial and patient co-morbidities, disease-related and prognostic factors, risks and benefits of surgery are all variables that have to be considered when planning cytoreductive nephrectomy.

There also appears to be a role of metastatectomy in a proportion of mRCC patients with limited sites of metastasis amenable to complete resection, prolonged period of disease-free interval and good performance status (13). Observational data suggests that, for the carefully selected patient, aggressive surgical resection followed by systemic therapy has the potential to prolong 5-year overall survival to 20–30% (14). Cytoreductive nephrectomy with palliative intent can also be offered to patients to control severe local or systemic symptoms such as intractable pain, bleeding and paraneoplastic manifestations such as hypercalcemia, hypertension, etc.

## Role of Radiation Therapy

RCC is considered to be an inherently radio-resistant tumour and, in mRCC, radiation therapy is primarily used for palliation of sites of painful metastasis (especially bone) and for treatment of brain metastases. Technological advancements in the field of radiation oncology which include image-guided radiotherapy and stereotactic radiosurgery are being explored in this setting and may expand the role of radiation therapy in the future.

## Systemic Therapy

There are a number of agents that are now approved for systemic therapy in mRCC. These include cytokine therapy, immunotherapy, chemotherapy and molecular targeted therapy. The molecular targeted agents include VEGF inhibitors like bevacizumab, TKIs such as sorafenib, sunitinib and axitinib, and the mammalian target of rapamycin (mTOR) inhibitors temsirolimus and everolimus. Prior to the FDA approval of the first TKI sorafenib in December 2005, cytokine therapy was most frequently used for mRCC. However due to the favorable side effect profile of the newer molecular targeted agents and their ease of administration, cytokine therapy usage has gradually decreased.

## Role of Cytokine Therapy and Immunotherapy

RCC is an immunologically driven malignancy and has therefore been amenable to immune manipulation. A number of immune potentiating strategies have been explored but only a few of them have been clinically successful. The two agents that have been heavily investigated are IFN alpha and interleukin-2 (IL-2). These two still have a role in the first line therapy of clear cell mRCC. High dose IL-2 is the only therapy that can produce durable complete or partial responses (14–28%) in a small subset of patients with metastatic, relapsed or unresectable RCC (15,16). Patient selection is critical as high dose IL-2 is associated with substantial toxicity and only patients with clear cell histology, an excellent performance status and minimal co-morbidity are likely to withstand this costly therapy and obtain benefit from it (17).

IFN alpha as a single agent has a response rate up to 15%, however the duration of response is usually short lived at approximately 4 months (18). More recently, it has been investigated in combination with molecular targeted agents with mixed results and increased toxicity. One successful combination that is now approved for first line treatment of clear cell mRCC is IFN alpha with bevacizumab.

## Role of Targeted Therapy

Several genetic and epigenetic changes are involved in the pathogenesis of RCC. Mutations in the von-Hippel-Landau (VHL) gene were first identified in hereditary RCC and then were also noted in 60–80% of sporadic RCC. The VHL protein is a tumor suppressor and this complex targets the hypoxia inducible factor (HIF) transcription factors which regulate important downstream targets such as VEGF, platelet-derived growth factor, and glucose transporter-1. However, a mutated VHL gene results in HIF accumulation and leads to a massive stimulation of growth factors which promote tumor growth and proliferation (19), including VEGF (20). The VEGF family ligands act via the VEGF receptor (VEGFR) to promote cell growth, proliferation, migration, chemotaxis and increase vascular permeability. This has a central role in cancer angiogenesis. VEGF inhibition was therefore investigated as a therapeutic strategy and initiated the development of several molecules, which ushered in the era of targeted therapy. Also involved in RCC pathogenesis is the mTOR pathway. The activation of mTOR complex results from the phosphatidylinositol 3-kinase (PI3K) pathway by growth factor receptors and further leads to downstream signal transduction responsible for the regulation of cell growth, proliferation, apoptosis, and metabolism of the cell. This pathway has also lent itself to therapeutic application in the mRCC patient and other solid malignancies.

Although it is simplistic to assume that the complex molecular cell signaling pathways can be permanently affected by a single molecule, the success of these targeted agents is proof of principle that at least temporarily the cancer cell can be effectively controlled.

## VEGF Inhibitors and TKIs

Bevacizumab is currently the only intravenous VEGF inhibitor in clinical use against RCC. It is a recombinant humanized monoclonal immunoglobulin G1 monoclonal antibody (mAb) that binds to VEGF extracellularly and prevents binding of VEGF to the VEGFR (primarily VEGFR-2), which leads to inhibition of its biologic activity. In the AVOREN study, bevacizumab in conjunction with IFN alpha as first line therapy for mRCC with clear cell histology prolongs median progression free survival (PFS) as compared to IFN alone (10.2 months versus 5.4 months; p=0.001), although there was no difference in OS (21,22). More than half the patients in both arms received at least one other line of therapy subsequently and this may have impacted the results of the OS analysis. In the Cancer and Leukemia Group B (CALGB) 90206 trial (23), the combination of bevacizumab and IFN alpha prolonged PFS but not OS as compared to IFN alpha alone. Bevacizumab is therefore approved in combination with IFN alpha for the first line therapy of clear cell mRCC. It has also been investigated as combination therapy with a TKI or mTOR inhibitor. However, as this led to increased toxicity without significant improvement in efficacy (24), this is currently not a favored approach.

Sunitinib is an oral multi-targeted TKI that binds to the intra-cellular domain of VEGFR, PDGF receptors (PDGFR) a and b, FLT-3, and other c-kit receptor tyrosine kinases. For treatment naïve patients with clear cell mRCC, sunitinib as compared to IFN alpha prolonged PFS (11 months versus 5 months; p =<0.001) (25) with higher overall response rate (ORR) in the sunitinib arm (31% versus 6%; p<.001). Sunitinib also resulted in prolonged OS (26.4 months versus 21.8 months; p=0.051), and patients on sunitinib had better quality of life (p= <0.0001).

Pazopanib is also a multi-targeted TKI of VEGFR, PDGFR-a/b, and c-kit. It was investigated for both treatment naïve and post-cytokine therapy clear cell mRCC. In comparison with placebo, it improved PFS (9 months versus 4.2 months; p=0.0001) (26). Both sunitinib and pazopanib are approved for first line treatment of mRCC, and the choice of therapy is often based on patient co-morbidities and keeping in consideration the side effect profile of these two drugs. The most severe adverse effects noted with sunitinib are neutropenia, thrombocytopenia, hyperamylasemia, diarrhea, hand-foot syndrome and hypertension. The side effects noted with pazopanib are diarrhea, hypertension, hair color changes, nausea, vomiting, anorexia, weakness, fatigue, hepatotoxicity, abdominal pain and headache. A large phase III non-inferiority trial with more than a thousand patients has demonstrated that the two drugs have similar efficacy. Pazopanib was found to be non-inferior to sunitinib with similar PFS and OS (27). Pazopanib has demonstrated greater patient acceptability as compared to sunitinib in a cross over trial where patients were exposed to both drugs after suitable wash-out period of each drug (28). Pazopanib was superior to sunitinib in health related quality of life measures evaluating fatigue, hand, foot and mouth soreness.

Sorafenib has a mechanism of action similar to sunitinib and, in addition to inhibiting VEGFR, FLT-3, c-kit, RET, PDGFR a and b, also inhibits serine and threonine and ras kinases. It was the first TKI approved for mRCC when it was shown to have an increase in response rate, prolongation of PFS and improvement in quality of life as compared to IFN alpha (29,30). At present, however, it is not routinely used in the first line setting, since the subsequent TKIs that were developed have demonstrated greater efficacy.

Axitinib is an extremely potent second generation TKI that selectively targets VEGFR and to a lesser degree PDGFRs and c-kit. For patients who have failed a prior systemic therapy, axitinib as compared to sorafenib has shown an improved response rate and PFS (6.7 months versus 4.7 months; p< 0.0001) (31). The benefit was more striking in patients who had received prior cytokine therapy. Outcome to second-line therapy was better when duration of first-line treatment was longer (32).

## mTOR Inhibitors

Temsirolimus administered intravenously was compared to IFN-alpha in a large multicenter phase III study of treatment naïve mRCC with poor prognosis (at least 3 of 6 poor risk predictors) and all histologies (33). Patients in the temsirolimus alone arm had longer OS (10.9 months versus 7.3 months; p = 0.008) and PFS (5.5 months versus 3.1 months; p<0.001) than those on IFN. It is important to reiterate that temsirolimus is the only drug that has approval for use in non-clear cell histology and poor prognosis mRCC.

Everolimus is approved in the second line setting for patients who have progressed after TKI/VEGF inhibitor therapy. In a randomized phase III trial, patients with mRCC who had progressed on sunitinib, sorafenib or both, or previous bevacizumab, IFN and IL-2, were assigned to everolimus or placebo (34). Median PFS was significantly longer in the everolimus arm as compared to the placebo arm (4.9 months versus 1.9 months, p= < 0.0001).

Although targeted therapy has changed our approach in treating patients with RCC, the evolution of the disease is such that these drugs cease to be effective after a limited period of time. Current studies are therefore evaluating combination regimens of targeted therapy with cytokine therapy or targeted therapies with different downstream signaling targets so as to prevent resistance and improve outcomes. This has not proven to be a very successful effort as the combinations studied have led to markedly increased toxicities without improving efficacy (35–40).

## Role of Chemotherapy

Chemotherapy has a very limited role in clear cell RCC, however there is some evidence for its use in the sarcomatoid variant which carries a very poor prognosis (41). Gemcitabine and doxorubicin combination has shown up to 16% response rates, 26% stable disease rate with a median OS of 8.8 months and PFS of 3.5 months (42). Gemcitabine has also been combined with capecitabine for metastatic progressive RCC with a response rate of 8.4 to 11% and median OS of 14.5 to 17.9 months (43–45).

There is also some literature supporting the use of combination chemotherapy in non-clear cell histologies especially for the renal medullary carcinoma and collecting duct carcinoma subtypes, both of which usually have advanced disease at presentation and, thereby, limited survival. Collecting duct carcinoma has histology similar to that of urothelial carcinoma, and gemcitabine combined with a platinum agent has shown response rates up to 26% with median PFS of 7.1 months and median OS of 10.5 months (46). Similar regimens have also been used in renal medullary carcinoma (47).

## Bone metastasis and supportive care

Supportive therapy, including pain relief and efforts to improve quality of life should be offered to all patients with metastatic RCC, as despite all advances it remains a terminal disease. Bone is one of the most frequent sites of metastasis, and can lead to significant skeletal related morbidity which includes bone pain, pathological fractures, spinal cord compression and hypercalcemia secondary to malignancy. Zoledronic acid significantly reduces the risk of these skeletal related events by approximately 61% and the therapeutic interventions for these events that may include palliative radiation and surgery (48). It also is believed to have antitumour activity and can potentiate the anti-cancer effects of targeted therapy thereby prolonging tumor control (49).

Denosumab is a fully human monoclonal antibody that binds to and neutralizes RANKL (receptor activator of nuclear factor-êB ligand), inhibits osteoclast function and prevents generalized bone resorption and local bone destruction. It is non-inferior (trending to superiority) to zoledronic acid in preventing or delaying first on-study skeletal-related events in various malignancies, including mRCC (50). It is easy to administer since it is given by the subcutaneous route and the absence of significant renal toxicity makes it a particularly attractive novel therapeutic option for patients with mRCC. The majority of these patients has undergone a nephrectomy in the past and may have compromised renal function.

## Conclusion

Multimodal, multi-disciplinary therapy is vital in the management mRCC, which remains a challenging and ultimately terminal disease. The management of this disease has continuously evolved through the years as increased scientific understanding of the molecular pathways involved in tumourigenesis led to development of various new drugs that target these distinct pathways. The dilemma that physicians face currently and questions that need to be answered on an urgent basis are appropriate selection and sequencing of the various novel agents, optimal dosing and combination strategies for improving efficacy and enhancing clinical outcomes.
